# Molecular characterization of adenosine monophosphate deaminase 1 and its regulatory mechanism for inosine monophosphate formation in triploid crucian carp

**DOI:** 10.3389/fphys.2022.970939

**Published:** 2022-08-30

**Authors:** Yonghua Zhou, Anli Zuo, Yingjie Li, Yu Zhang, Zilin Yi, Dafang Zhao, Jianzhou Tang, Fufa Qu, Shenping Cao, Zhuangwen Mao, Junyan Jin, Zhen Liu

**Affiliations:** ^1^ Hunan Provincial Key Laboratory of Nutrition and Quality Control of Aquatic Animals, Department of Biological and Environmental Engineering, Changsha University, Changsha, China; ^2^ State Key Laboratory of Freshwater Ecology and Biotechnology, Institute of Hydrobiology, Chinese Academy of Sciences, Wuhan, China

**Keywords:** AMPD1, triploid crucian carp, glutamate, regulatory, inosine monophosphate

## Abstract

Inosine monophosphate (IMP) is the main flavoring substance in aquatic animal, and adenosine monophosphate deaminase1 (*AMPD1*) gene is a key gene in IMP formation. At present, the research on the mechanism of *AMPD1* regulating IMP formation in aquatic animal is still blank. In this study, in order to study the mechanism of *AMPD1* regulating IMP formation in fish, the full open reading frame (ORF) of *AMPD1* which was 2160bp was obtained for the first time in triploid crucian carp (*Carassius auratus*). It encoded 719 amino acids with a molecular mass of 82.97 kDa, and the theoretical isoelectric point value was 6.31. The homology analysis showed that the homology of triploid crucian carp and diploid *Carassius auratus w*as the highest, up to 99%. And the phylogenetic tree showed that triploid crucian carp was grouped with diploid *Carassius auratus*, *Culter alburnus*, and *Danio rerio*. And real-time fluorescence quantitative results showed that *AMPD1* was expressed specifically in muscle of triploid crucian carp (*p* < 0.05). The results of detection the localization of *AMPD1* in cells indicated that the *AMPD1* was mainly localized in cytoplasm and cell membrane. Further, we examined the effects of glutamate which was the promotor of IMP formation on the expression of *AMPD1* and the formation of IMP *in vivo* and *in vitro* experiments, the results showed that 3% glutamate and 2 mg/ml glutamate could significantly promote *AMPD1* expression and IMP formation in triploid crucian carp muscle tissue and muscle cells (*p* < 0.05). Then we inhibited the expression of *AMPD1 in vivo* and *in vitro* experiments, we found the formation of IMP in muscle tissue and muscle cells of triploid crucian carp all were inhibited and they affected the gene expression of AMPK-mTOR signaling pathway. The all results showed that *AMPD1* mediated glutamate through AMPK-mTOR signaling pathway to regulate the formation of fish IMP.

## Introduction

Fish and other aquatic products with its delicate and delicious meat, rich in protein and low-fat characteristics, become consumers love the nutritious food. However, with the improvement of people’s living standards, the requirements for the umami quality of fish are getting higher and higher (Xiao et al., 2021; Zhu et al., 2021). At present, modern aquaculture industry is in a period of rapid development, people always feel that the quality of artificial aquaculture fish meat is also declining (Itoh and Kimura, 2002; Hao et al.,2021; Surowka et al., 2021). Therefore, how to improve the meat quality of cultured fish, make it more delicious and improve its market economic value is a hot issue in aquaculture research. Umami is an important indicator to measure the quality of aquatic products, among which inosine monophosphate (IMP) is the main umami substance in aquatic products, and IMP is an important factor to affect the umami quality of fish and consumer preference (Hu et al., 2015; Bonagurio et al., 2022). The enzyme encoded by *AMPD1* gene is the key enzyme that catalyzed the cleavage of adenosine monophosphate (AMP) to generate IMP, that is, *AMPD1* gene plays a decisive role in the formation of IMP(Norman et al., 1998; Norman et al., 2001; Thebault et al., 2010; Hu et al., 2015). At present, studies on *AMPD1* were mainly focused on higher animals such as humans, and most of the studies on humans were medical studies (Norman et al., 2001; Cheng et al., 2014). For example, *AMPD1* deficiency could produce symptoms of metabolic myopathy. And research on *AMPD1* gene in livestock, poultry and aquatic animals mainly focused on cloning *AMPD1* gene sequence and analyzing the relationship between *AMPD1* gene polymorphism and IMP content (Yu et al., 2021; Zhang et al., 2021; Zhang et al., 2019). In general, all studies had only studied the correlation between *AMPD1* gene polymorphism and IMP content. But the research on the mechanism of *AMPD1* regulating IMP formation was still blank. Previous study had shown that *AMPD1* also played an important role in energy metabolism of the mammalian body. And it could affect AMP content in the process of regulating IMP formation in mammal (Tandelilin et al., 2015). And existing research had shown that AMPK (AMP activated protein kinase) enzyme activity could be rose by AMP(Bausch-Jurken and Sabina, 1995; Admyre et al., 2014). AMPK is widely present in various eukaryotic cells and is an important cell energy sensor. Studies in mammals have also shown that AMP level changes can regulate biological energy metabolism by altering the activity of AMP-activated protein kinase (AMPK). This because AMPK can be activated by sensing changes in the ratio of AMP: ATP and ADP: ATP, restoring energy balance by inhibiting anabolic processes that consume ATP and promoting catabolic processes that produce ATP (Hardie, 2008; Plaideau et al., 2014). In other words, when AMPK is activated, it can increase ATP production and decrease ATP consumption through increasing glycolysis and inhibiting protein synthesis, thus affecting the formation of IMP. And mTOR is the downstream signaling pathway of AMPK. The catalytic domain of mTOR contains different phosphorylation sites, including AMPK in the upstream and p70S6K1 in the downstream. Previous study has shown that AMPK directly phosphorylates raptor and blocks the ability of the mTOR kinase complex to phosphorylate its substrates. And one of the mTOR targets, phosphorylates ribosome S6 protein (p70S6K1) can enhance the translation function of pyrimidine mRNA, thereby affecting ATP metabolism and the formation of IMP. These studies suggest that *AMPD1* may affect ATP metabolism through AMPK-mTOR and thus IMP formation (Plaideau et al., 2012; Tandelilin et al., 2015). However, the specific regulatory mechanism is not clear. Studies have shown that dietary amino acid composition can affect the content of muscle IMP(Leclerc and Rutter, 2004; Li et al., 2021). Glutamate is an important flavor amino acid, and its umami intensity is 30 times higher than that of the same amount of glutamate when the ratio of glutamate and IMP is 1:1. The results showed that dietary glutamate could increase the IMP content in muscle of prawn. In addition, studies on yellow-feathered broilers showed that dietary glutamate could improve the content of flavor amino acids in muscle and improve meat flavor (Jiang et al., 2015; Jiang et al., 2016; Jiang et al., 2017; Yan et al., 2018). In fish studies, it was found that dietary glutamate could improve the muscle fiber structure and muscle texture of grass carp, and increase the content of IMP in muscle (Pal Choudhuri et al., 2016; Jiang et al., 2017). But the molecular mechanism of how glutamate affected IMP formation was not clear. Therefore, we focus on the research of the mechanism of *AMPD1*-mediated glutamate regulating triploid crucian carp IMP formation. In a word, umami is one of the core indicators of fish quality traits. *AMPD1* plays an important role in the regulation of IMP formation, but the molecular basis of *AMPD1* regulation of IMP deposition in fish muscle remains unclear (Li et al., 2020; Pal Choudhuri et al., 2016). In this study, we took triploid crucian carp as the research object, and adopt molecular and cell biology technology and nutritional regulation technology to carry out a systematic study on the function and regulation mechanism of *AMPD1*, a key gene in the process of fish muscle IMP formation. Firstly, the molecular characteristics and tissue expression characteristics of *AMPD1* gene in triploid crucian carp were determined by using molecular biology techniques. Then, the regulation effects of glutamate which was the promotor of IMP formation on the expression of *AMPD1* and the formation of IMP were determined *in vivo* and *in vitro* experiments. Further, the mechanism of *AMPD1* on IMP formation through AMPK-mTOR signaling pathway was revealed through *in vivo* and *in vitro* experiments. Finally, the molecular mechanism of interaction between *AMPD1*, glutamate, AMPK-mTOR signaling pathway and muscle IMP was analyzed. Through the above studies, we hoped to clarify the mechanism of *AMPD1*-mediated glutamate regulating triploid crucian carp IMP formation, which would lay a foundation for in-depth analysis of the molecular mechanism of fish IMP formation, and provide theoretical basis and technical support for fish meat quality improvement and further development and utilization of fish resources. Therefore, this study would provide a theoretical basis for establishing a reasonable feeding strategy for cultured fish and improving the umami quality of cultured fish, and put forward a new idea for in-depth study on accurate nutrition regulation of fish quality traits.

## Materials and methods

### Animals and sample preparation

All healthy triploid crucian carp (300.0 ± 2.5 g) were purchased from the Hunan Institute of Aquatic Science. And they were stored in a 300 L round glass fiber tank in the indoor circulating fresh water system. All fishes were adapted to the aquaculture conditions for 2 weeks, and the water temperature was controlled at (27.0 ± 0.5)°C and recorded daily. The experimental photoperiod was regularly controlled for a 12 h light-dark cycle (light on 8:00 a.m.). And triploid crucian carp individuals were randomly collected and anesthetized with 20 mg/L 2-phenoxyethanol (Sigma-Aldrich) before dissecting triploid crucian carp to obtain tissue samples. And the tissue samples (the brain, gut, kidney, liver, muscle, heart, and spleen) were collected for *AMPD1* gene cloning and tissue distribution analysis. And they were stored at −80°C for reserve.

### Cloning of triploid crucian carp adenosine monophosphate deaminase1 cDNA open reading frame region

Trizol reagent (TakaRa) was used to extract total RNA from muscle samples of triploid crucian carp. And OD260/280 ratio of total RNA was between 1.90 and 1.95. Then we used the total RNA to reverse transcription to get cDNA. The first strand of cDNA synthesized by AMV reverse transcriptase (Fermentas). We reference the *AMPD1* gene sequence of *Carassius auratus* for homologous cloning. Then we designed the primers of *AMPD1* clone by using primer design software Primer5.0 (Table1). And we cloned the ORF-containing target fragment of *AMPD1* by PCR kit (TakaRa), then we used DNA purification kit (TakaRa) to purify ORF-containing DNA fragment, then it was linked to pMD19-T plasmid (TakaRa), mixed and placed in a constant temperature incubator 16°C overnight, the products were transformed into competent *escherichia coli* cells for culture the next day, and the positive clones were verified by PCR. 200 μl of positive clone solution consistent with the size of the target DNA fragment was selected and sent to Shanghai Boshang Biotechnology Co., LTD. for sequencing.

**TABLE 1:** The sequences of the designed primers used in this study.

Primer name	Sequence (5′→3′)	Comment	Genebank
*AMPD1*-F	TTC​TAC​CGA​AAG​AGT​GAT​AGT	CDS Cloning	XM_026245035.1
*AMPD1*-R	TCA​TGG​GCT​TCA​CAT​TAA​CGA		
*AMPD1*-F1	ATC​TAT​GGC​TGT​AAC​CCT​AAT	qRT-PCR	MW435570.1
*AMPD1*-R1	ATG​TCA​TAG​ATT​CTG​GGC​ACT		
*AMPK*-F1	AAG​TCC​AAG​CAT​CTC​GGT​GTC	qRT-PCR	MK294331.1
*AMPK*-R1	TGG​GTT​CTT​CCT​CCG​CAC​T		
*mTOR*-F1	CCA​AAG​AGA​TGC​AGA​AGC​CAC​A	qRT-PCR	XM_026262450.1
*mTOR*-R1	CTC​TCT​CAT​ACG​CTC​TCC​CT		
*p70S6K1*-F1	CCT​CCT​CAT​GAC​ACC​CTG​CT	qRT-PCR	XM_026285746.1
*p70S6K1*-R1	TCT​TCT​GGT​CCG​TTG​GCA​AA		
*β-actin*-F	CCT​TCT​TGG​GTA​TGG​AGT​CTT​G	qRT-PCR	MN184794.1
*β-actin*-R	AGA​GTA​TTT​ACG​CTC​AGG​TGG​G		

F, forward primer, R, reverse primer.

### Bioinformatics analysis of triploid crucian carp adenosine monophosphate deaminase1 open reading frame region

DNAMAN software was used to splice the cDNA sequences, and NCBI BLAST Online software (https://blast.ncbi.nlm.nih.gov/Blast.cgi) was used to query the homology similarity of *AMPD1* gene sequences among the species in the database. The open reading frame (ORF) finder tool on NCBI (https://www.ncbi.nlm.nih.gov/orffinder/) was used to analyze the ORF of the *AMPD1* gene sequence of triploid crucian carp and further derive the amino acid sequence of the gene. Protein analysis of basic physical and chemical parameters and structure using online website (https://web.expasy.org/protparam/) was analyzed; and MEGA 5.1 was used to construct a phylogenetic tree based on Neighbor Joining method with 1,000 bootstrap replicates.

### Adenosine monophosphate deaminase1 gene eukaryotic expression vector construction

Based on the sequence of its open reading frame the *AMPD1* gene was cloned from the cDNA library of the triploid crucian carp muscles using PCR amplification with the sense primer 5′-GAA​ATG​GAG​GAT​TCG​GCC​TCG​GCC-3′ and the antisense primer 5′-GGA​TCCTTA​GTC​ACC​AAT​CAC​GCC​CTC​T-3' (EcoRI and BamHI restriction sites were added at the 5'end of the primer). The PCR amplification conditions were as follows: 95°C (30 s), 58°C (1 min) and 72°C (2 min), 30 cycles. The *AMPD1* cDNA was cloned into the eukaryotic expression vector pEGFP-C3(Clontech) which contained the same restriction sites in its multiple cloning sites. The resulting recombinant containing *AMPD1* cDNA was confirmed by sequencing and named pEGFP-C3-*AMPD1*.

### Transient transfection and subcellular localization of adenosine monophosphate deaminase1 fusion protein

293T-cells were grown in Dulbecco’s Modified Eagle Medium (DMEM)medium (Invitrogen, CA, United States) with the addition of 10% fetal calf serum and 500 µ/ml penicillin, 500U/ml streptomycin. The construction of expression plasmid pEGFP-C3-*AMPD1* was then transfected into the 293T-cells after the second day of cell passage. Briefly, the transient transfection of 293T-cells was performed using Lipofectamine (Invitrogen, CA, United States) according to the manufacturer’s instructions. Approximately 1.0 µg of cDNA per 150,000 cells was used in 6-well plates. Then cell DNA was stained with hoechst33342 for 30 min after transfection for 48 h, then cell fluorescence was imaged using Zeiss LSM510 confocal microscope (Carl Zeiss AG, Oberkochen, Germany).

### Quantitative real-time PCR analysis

All triploid crucian carp tissue total RNA was isolated using Trizol reagent (Invitrogen, Carlsbad, CA). And OD260/280 ratio of total RNA was detected by spectrophotometry (BioPhotometer, Eppendorf). Then the total RNA (1.0 μg) from each sample was used to synthesize the first strand of cDNA. And we used AMV reverse transcriptase (Fermentas, Vilnius, Lithuania) for cDNA synthesis according to the manufacturer’s instructions. The mRNA levels of *AMPD1*, *AMPK*, *mTOR*, and *p70S6K1* were determined with a Prism 7500 Sequence Detection System (Applied Biosystems, Foster City, CA, United States). And β-actin was used as an endogenous control. The PCR primers were shown in Table1. Amplifications were performed in triplicate in a total volume of 10.0 µl reactions included 1.0 µl cDNA, 0.4 µl forward and reverse primer, 5.0 µl of SYBR Premix ExTaq polymerase (TaKaRa) and 3.2 µl ddH_2_O.And the PCR conditions were as follows: 95°C for 30 s followed by 40 cycles of 95°C for 3 s, 60°C for 25 s and 72°C for 10 s. In addition, negative template control reactions were used and dissociation curves were created to rule out DNA and dimmer contamination. The relative expression was calculated by using the 2^−ΔΔCt^ method.

### The effects of glutamate on the relative mRNA expression of adenosine monophosphate deaminase1 and the inosine monophosphate content *in vivo* and *in vitro*

*In vivo* experiment, in order to analyze the effects of different proportions of dietary glutamate additives on the IMP content and the relative mRNA expression of *AMPD1*. In this experiment, fish meal, soybean meal, rapeseed meal and cottonseed meal were used as main protein sources, and fish oil and soybean oil were used as lipid sources to prepare isonitrogenous, isolid and isolic basal diets. On this basis, 0.0%, 1.0%, 1.5%, 2.5%, and 3.0% glutamate (purity ≥98%, Xi ‘an Tiankang Biotechnology Co., Ltd., China) were added, respectively. All raw materials were crushed through a 60-mesh sieve, and a 1.5 mm particle size pellet feed was made by a ring mold feed machine (SZLH200, Jiangsu Zhengchang Group Co., Ltd). After drying, it was sealed and stored in a freezer at −20°C for later use. Feed raw material composition and nutrient content are shown in Table 2. Triploid crucian carp (300.0 ± 6.5) g was adopted in the experiment from Hunan Fisheries Research Institute. And the experimental fish were domesticated for 2 weeks, and the domesticated feed was the basic feed. During domestication, basal diet was fed at 8:30 and 14:30, respectively. Before the experiment, the fish were fasted for 24 h, and the healthy and uniform individuals were selected. After weighing, they were randomly placed into 15 fish tanks in the fish room of the laboratory (water temperature: 27.0 ± 0.5°C). There were five treatments in total, with 30 parallel fish in each treatment and 450 fish in each parallel. The experiment lasted for 70 days. During the experiment, fish were fed at 7:30 and 17:30 to apparent satiation. The laboratory tested the water temperature once a day and recorded the daily food intake of each tank of experimental fish. The dissolved oxygen content is always maintained above 6.5 mg/L, the ammonia nitrogen concentration is less than 0.5 mg/kg, and the pH range is 7.0–7.8. The experiment was illuminated by fluorescent lamp (8:00–20:00).

**TABLE 2:** Formula and nutritional composition of experimental diets.

Ingredients	Glutamate (Glu)levels				
	0.0%	1.0%	1.5%	2.5%	3.0%
Glu	0.00	1.00	1.50	2.50	3.00
Fish meal	2.00	2.00	2.00	2.00	2.00
Soybean meal	28.00	28.00	28.00	28.00	28.00
Rapeseed meal	18.00	18.00	18.00	18.00	18.00
Cottonseed meal	10.80	10.80	10.80	10.80	10.80
Flour plant	16.00	16.00	16.00	16.00	16.00
Fish oil	2.00	2.00	2.00	2.00	2.00
Soya-bean oil	2.50	2.50	2.50	2.50	2.50
α-starch	4.00	4.00	4.00	4.00	4.00
Corn starch	4.00	4.00	4.00	4.00	4.00
Choline	0.11	0.11	0.11	0.11	0.11
Mineral premix^a^	3.00	3.00	3.00	3.00	3.00
CMC	3.00	3.00	3.00	3.00	3.00
Cellulose	6.59	5.59	5.09	4.09	3.59
Nutrition level					
Crude protein	31.2	32.02	32.52	33.52	34.02
Crude lipid	6.03	6.03	6.03	6.03	6.03
Ash	5.66	5.78	5.85	5.55	5.62
Gross energy (MJ kg^−1^)	17.45	17.62	17.70	17.87	17.95

Note: Mineral premix^a^ (mg kg^−1^ diet): NaCl, 250.0; NaH_2_PO4 2H_2_O, 6250.0; MgSO_4_ 7H_2_O, 4077.8; KH_2_PO_4_, 8000.0; CaH_2_PO_4_ 2H_2_O, 3825.3; C_6_H_10_CaO_6_ 5H_2_O, 875.0; FeSO_4_ 7H_2_O, 1143.1; ZnSO_4_ 7H_2_O, 89.0; MnSO_4_·H_2_O, 30.7; CuSO_4_ 5H_2_O, 7.75; CoSO_4_ 7H_2_O, 0.46; KI, 0.75; Na_2_SeO_3_,0.30; Corn starch, 499.9.

*In vitro* experiment, the fish muscle tissue was rapidly separated from triploid crucian carp (4.0 ± 0.5) g after anesthetized with 20 mg/L 2-phenoxyethanol. Then the fish muscle tissues were cut into pieces by using scissors. After washed three times with PBS containing 500U/ml penicillin (Gibco, United States) and 500 µg/ml streptomycin (Gibco, United States), minced fish muscle tissues were seeded in cell culture dishes with Dulbecco’s Modified Eagle Medium/Nutrient Mixture F-12 (DMEM/F12) containing 15% FBS (Gibco, United States), 100U/ml penicillin, 100 µg/ml streptomycin and 15 ng/ml fibroblast growth factor (FGF, PEPROTECH, United States) 5% CO_2_ at 28°C. Afterward, culture medium was replaced with fresh cell culture medium by half every 2 days. Subculture was carried out at a split ratio of 1:2 when primary cell cultures grew to above 90% of confluence. When the fish muscle cells passed on to the third generation, then the muscle cells were inoculated into 6-well plates and randomly uniformly divided into five groups of 3 wells. After passaging, when the cells adhered well and were normally extended. The original cell culture medium was aspirated using a sterile pipette and gently washed with 2 ml PBS for one time. Then complete cell medium containing different concentrations of glutamate (0, 1, 1.5, 2, and 2.5 mg/ml) were added to each group of cells. After 24 h of culture, removed the cell culture medium and collected the cell to analyze the effects of different proportions of glutamate additives on the relative mRNA expression of *AMPD1* and the IMP content.

### Intraperitoneal injection adenosine monophosphate deaminase1 Inhibitor *in vivo* and adenosine monophosphate deaminase1 inhibitor addition experiment *in vitro*

For *in vivo* experiments, triploid crucian carp (300.0 ± 5.5) g were selected for intraperitoneal injection of AMPD1 inhibitor pentostatin (MedChemExpress, United States) at different concentration *in vivo* after 2 weeks of domestication. According to the weight of our experimental triploid crucian carp, we conducted the experiment at the concentration gradient of pentostatin addition. Then the experiment was performed by injecting 200 µl of phosphate-buffered saline (PBS) as the control group and injected 200 µl pentostatin at doses of (0.4, 0.8, 1.2, and 1.6) mg/kg into the experimental group. The intraperitoneal injection experiments were designed with 3 fish repeats and set to 3 repetitions per group. The experiment was carried out in the indoor circulation culture system of the Aquaculture Base of the College of Biotechnology, Changsha University. At 48 h and 72 h after intraperitoneal injection, samples were collected to detect relative *AMPD1*, *AMPK*, *mTOR*, and *p70S6K1* mRNA expression by quantitative real-time PCR.

For *in vitro* experiments, after fish muscle cell subculture, the seven-generation triploid crucian carp muscle cells were inoculated into 6-well plates and randomly divided into 4 groups of 3 wells. After passaging, when the fish muscle cells adhered well and were normally extended. The original fish muscle cell culture medium was aspirated using a sterile pipette and gently washed with 2 ml PBS for one time. Then complete cell medium containing different concentrations of pentostatin (0, 20, 40, and 60 µM) were added to each group of cells. After 16 h of culture, each group of the cell culture medium was removed, and the fish muscle cells were collected with a scraper and suspended with PBS, and gently washed with 2 ml PBS. Then the cells were collected by centrifugation 300 *g* for 3 min. Finally, all cell RNA was extracted after cell precipitation was obtained, and the relative mRNA expression levels of *AMPD1*, *AMPK*, *mTOR*, and *p70S6K1* were detected by quantitative real-time PCR.

### Detection of inosine monophosphate content by high performance liquid chromatography

Each fish group took muscle tissue (0.4 ± 0.05) g and put it into 200 ml beaker, added 90 ml water, then put the beaker into a constant temperature water bath, kept the water temperature at 95°C, boiled 5 min.The solution was filtered by 0.45 μm filter membrane, and then the sample was analyzed. And the chromatography was performed on an Agilent Zorbax SB-C18 column (150 mm × 4.6 mm, 5 μm). The mobile phase was methanol-50 mmol/L ammonium acetate aqueous solution (5:95, V/V). The column temperature was 30°C, and the flow rate was 1.0 ml/min. The injection volume was 10.0 μl. The detection wavelength was 254 nm, and the machine (Agilent 218, United States) was cleaned with water (ultra-pure water was filtered twice with 0.45 μm filter membrane) and methanol (chromatographic pure) before running the program for 7 min. Then, the machine was washed with methanol for 30 min. When no peak shape appeared, the machine was washed with methanol-50 mmol/L ammonium acetate solution until no peak appeared. Standard sample concentration: using 10.0 mg/ml IMP standard concentration sample with 0.02 mg/ml, 0.04 mg/ml, 0.06 mg/ml, 0.08 mg/ml, 0.10 mg/ml, 0.12 mg/ml of standard sample. Determination of the standard curve: determine the peak shape and area of sample IMP according to the peak of the standard sample, export the data, make statistics, and saved the data in Excel for later data calculation. After the determination, cleaned the machine and injection place with water and methanol.

### Statistical analyses

All experiments were repeated at least three times. Statistical analysis was performed by one-way ANOVA in the SPSS19.0 software (Chicago, IL, United States) statistical software package. The values are shown as mean ± SD (*n* = 3), and *p* < 0.05 was considered statistically significant.

## Results

### Cloning and sequence analysis of adenosine monophosphate deaminase1 gene in triploid crucian carp

**FIGURE 1:**
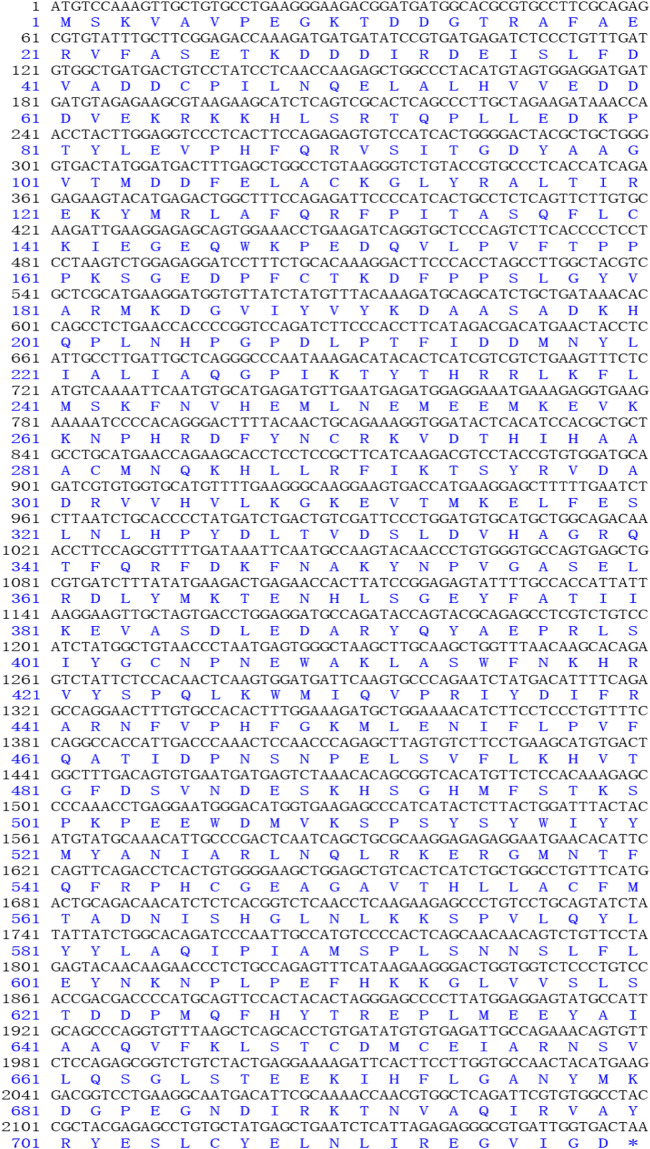
The open reading frame (ORF) and amino acid sequences of AMPD1.

**FIGURE 2:**
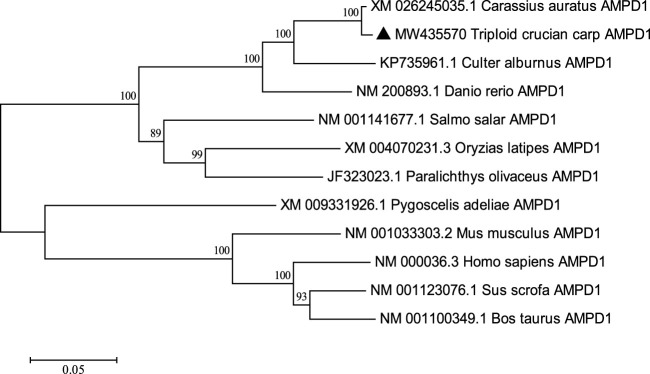
Adjacency phylogenetic tree based on AMPD1 amino acid sequences of different species. Node values represent the percent bootstrap confidence level derived from 1000 replicates. The bar (0.05) indicates the genetic distance.

The complete cDNA sequence of *AMPD1* was isolated from the muscle tissue cDNA library of triploid crucian carp and deposited in GenBank with accession number MW435570. The ORF sequence of the *AMPD1* gene was 2,160 bp, encoding 719 amino acids (Figure 1). Expasy prediction showed that the molecular weight of the AMPD1 protein was 82.97 kDa, the theoretical isoelectric point value was 6.31. BLAST analysis of amino acid homology of AMPD1 protein in triploid crucian carp revealed that AMPD1 amino acid sequence of triploid crucian carp was similar to that of other species published in NCBI *Carassius auratus* (XP_026100821.1) with 99% homology; Its homology with *Danio rerio* (NP_957187.1) was 92%. And its homology with *Culter alburnus* (AKQ62701.1), *Oncorhynchus mykiss* (XP_004070279.1) and *Salmo salar* (NP_001135149.1) was 91%, 81%, and 81% respectively (Table 3). Phylogenetic tree of AMPD1 amino acid construction of 12 species showed that the triploid crucian carp and diploid crucian carp had the closest genetic relationship and belong to the same branch (Figure 2).

**TABLE 3:** The amino acid sequence of AMPD1 was compared with that of other species.

Protein name	Accession no.	Amino acid comparison	
		Similarity (%)	Identity (%)
*Homo sapiens* AMPD1	NP_000027.3	81	68.2
*Bos taurus* AMPD1	NP_001093819.1	81	69
*Sus scrofa* AMPD1	NP_001116548	82	69
*Mus musculus* AMPD1	NP_001028475.2	82	69
*Pygoscelis adeliae* AMPD1	XP_009330198.1	70	82
*Danio rerio* AMPD1	NP_957187.1	92	95
*Carassius auratus* AMPD1	XP_026100821.1	99	99
*Salmo salar* AMPD1	NP_001135149.1	81	88
*Oryzias latipes* AMPD1	XP_004070279.1	81	89
*Paralichthys olivaceus* AMPD1	XP_019944239.1	82	90
*Culter alburnus* AMPD1	AKQ62701.1	91	95
Triploid crucian carp AMPD1	MW435570	—	—

### The expression characteristics of adenosine monophosphate deaminase1 in different tissues of triploid crucian carp

**FIGURE 3:**
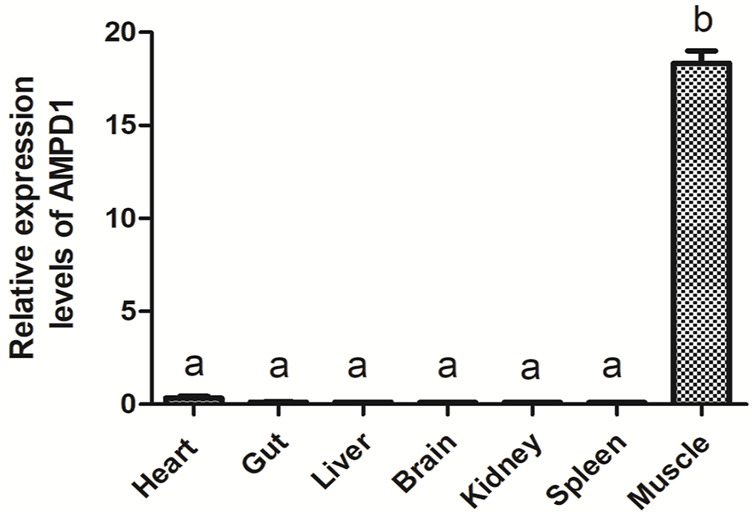
The relative expression levels of AMPD1 gene in different tissues of triploid crucian carp were analyzed by real-time fluorescence quantitative PCR. Error bars represent the means ± SD; *n* = 3. *p* < 0.05.

Quantitative real-time polymerase chain reaction (qPCR) was carried out to detect the mRNA expression level of *AMPD1* in different tissues of triploid crucian carp, including the heart, gut, liver, brain, kidney, spleen, muscle. The results showed that triploid crucian carp *AMPD1* gene expression were different in different organizations, and *AMPD1* was expressed specifically in muscle of triploid crucian carp (*p* < 0.05), while the expression level of *AMPD1* in heart, intestine, liver, brain, kidney and spleen was extremely low (Figure 3), indicating that *AMPD1* played an important role in maintaining muscle quality and flavor.

### Detection of the expression and localization of adenosine monophosphate deaminase1 in cells

**FIGURE 4:**
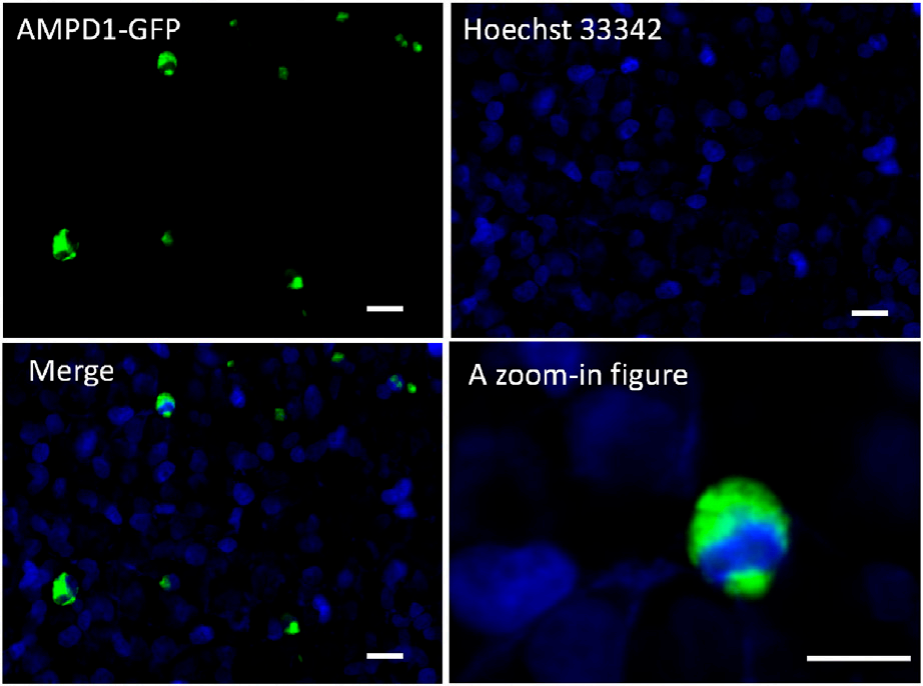
Localization analysis of AMPD1 in cells. Scale bars represent 50 µm.

To determine the cellular localization of recombinant *AMPD1*, the expression construct pEGFP-C3-*AMPD1* was transfected into 293T cells, and cell DNA was stained with hoechst33342 for 30 min after transfection for 48 h, then cell fluorescence was imaged using Zeiss LSM510 confocal microscope (Carl Zeiss AG, Oberkochen, Germany). The results indicated that the AMPD1 protein was mainly localized in cytoplasm and cell membrane (Figure 4). These results suggested that *AMPD1* played a regulatory role in IMP formation in cytoplasm and cell membrane.

### The effects of glutamate on the relative mRNA expression of adenosine monophosphate deaminase1 and fish inosine monophosphate content *in vivo* and *in vitro*

**FIGURE 5:**
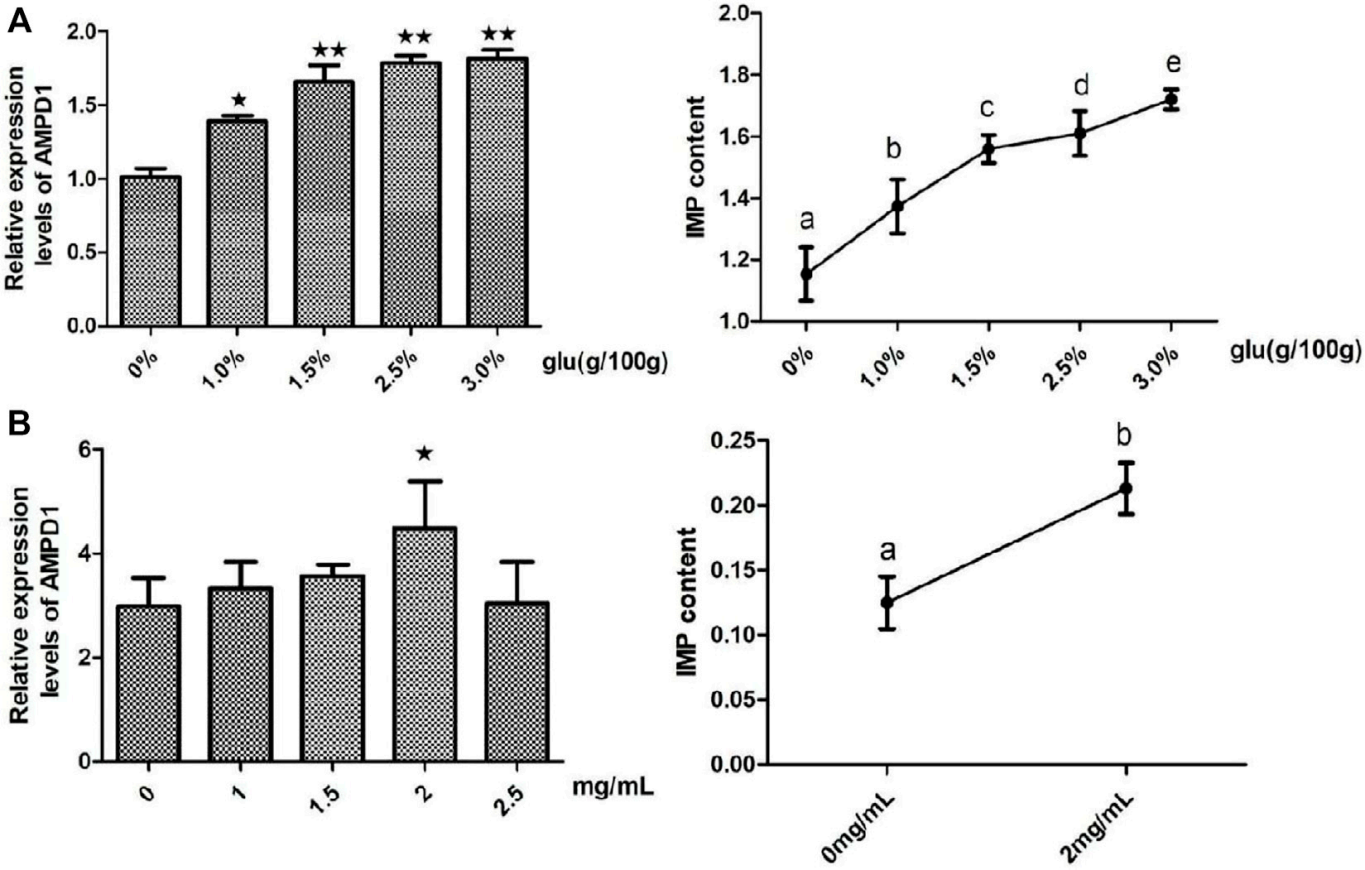
Effects of glutamate (Glu) on the relative mRNA expression of AMPD1 and the IMP content *in vivo* **(A) **and *in vitro* **(B)**. Error bars represent the means ± SD; *n* = 3. *p* < 0.05.

In order to clarify the regulation effects of glutamate (Glu) which was the promotor of IMP formation on the expression of *AMPD1* and the formation of IMP. We detected the effects of different concentrations of glutamate (Glu) on the relative mRNA expression of *AMPD1* and the IMP content *in vivo* and *in vitro*, and the results showed that different concentrations of Glu could promote the relative mRNA expression of *AMPD1* and increase IMP content in muscle, and the concentration of 3% Glu could promote the greatest effect on the relative mRNA expression of *AMPD1* and increase IMP content in muscle *in vivo* (Figure 5A). And we found that the use of 2 mg/ml Glu could promote the relative mRNA expression of *AMPD1* and increase IMP content in fish muscle *in vitro* (Figure 5B).

### Effects of AMPD1 Inhibitor Injection on Fish IMP Content and the Relative mRNA Expression of AMPK-mTOR Signaling Pathway Related Genes for 48 h and 72 h *in vivo*

**FIGURE 6:**
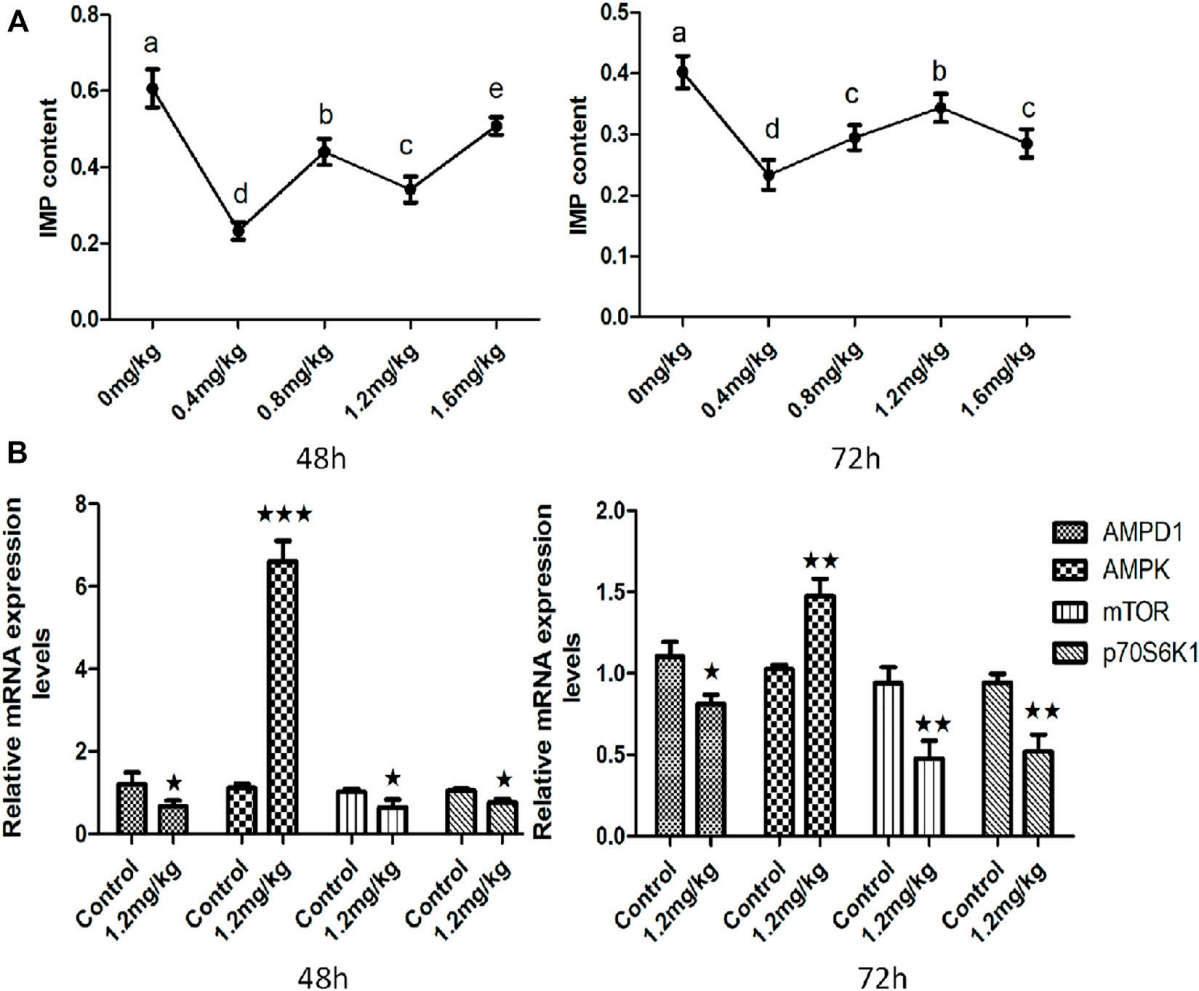
Effects of AMPD1 inhibitor injection on IMP content and the relative mRNA expression of AMPK-mTOR signaling pathway related genes for 48 h and 72 h *in vivo*. **(A)** IMP content; **(B)** the relative mRNA expression of AMPK-mTOR signaling pathway related genes. Error bars represent the means ± SD; *n* = 3. *p* < 0.05.

In order to further clarify the molecular mechanism of *AMPD1* regulation of IMP formation, we verified it by injecting AMPD1 inhibitor at the body level. Firstly, (0.4 ± 0.05) g muscle tissue of the control group and the experimental group were respectively for IMP detection after AMPD1 inhibitor injected for 48 h and 72 h. The results showed that different concentrations of AMPD1 inhibitor (0, 0.4, 0.8, 1.2, and 1.6 mg/kg) had different effects on the formation of IMP, but both low concentration and high concentration of AMPD1 inhibitor inhibited the formation of IMP compared with the control (Figure 6A). Next, we analyzed the effects of injection of AMPD1 inhibitor (0, 1.2 mg/kg) after 48 h and 72 h, the expression of *AMPK*, *mTOR*, and *p70S6k1* was detected and analyzed by Q-PCR. The results showed that the injection inhibitor had a down-regulating effect on mRNA expression levels of *AMPD1*, *mTOR* and *p70S6k1* genes after 48 h and 72 h. But it promoted *AMPK* expression (Figure 6B).

### Effects of adenosine monophosphate deaminase1 inhibitor on the inosine monophosphate content and AMPK- mTOR signaling pathway related genes *in vitro*

**FIGURE 7:**
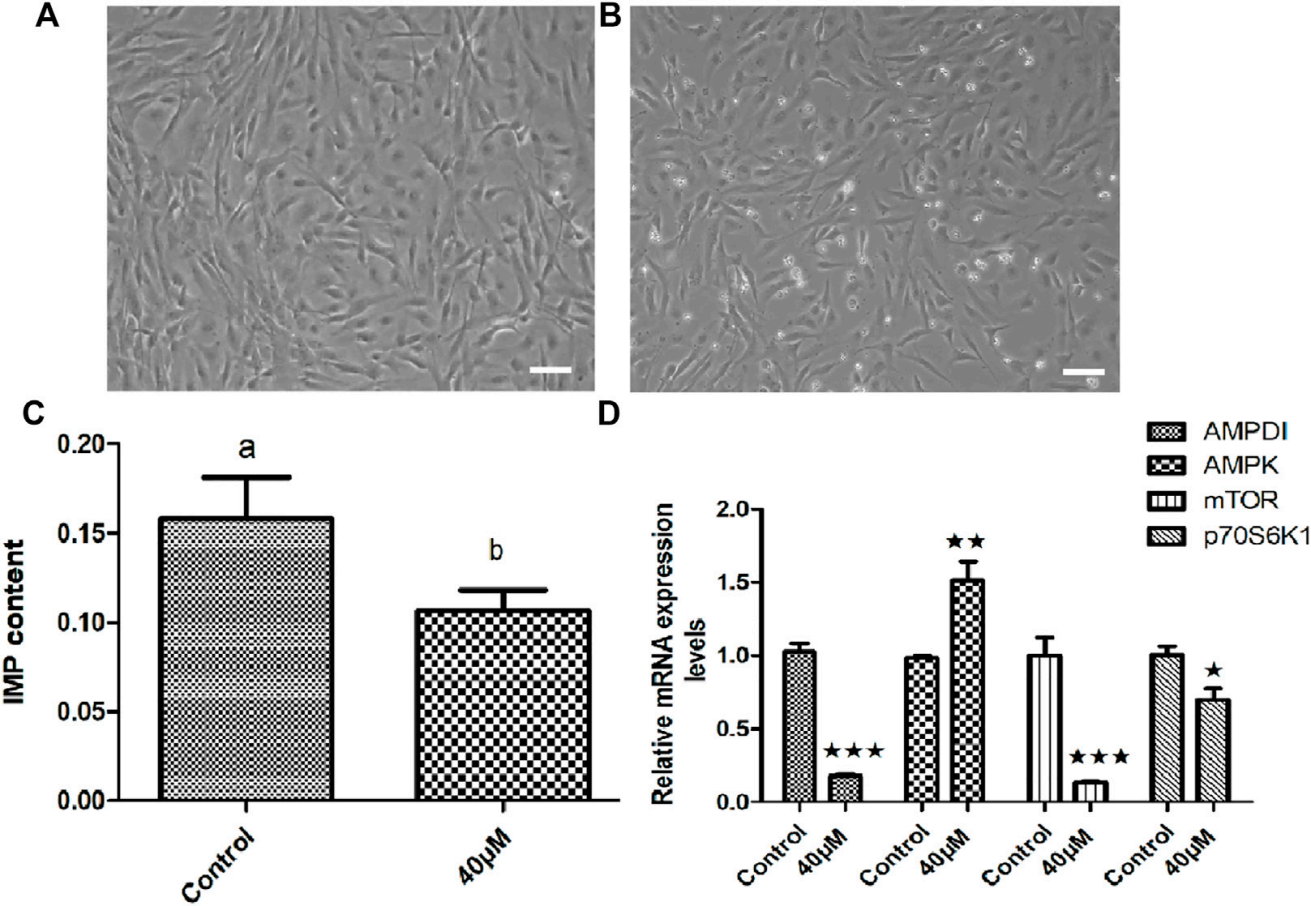
Effects of 40 μM AMPD1 inhibitor on the IMP content and AMPK-mTOR signaling pathway related genes *in vitro*. **(A)** the sixth generation of muscle cells, **(B)** the sixth generation of muscle cells treated with 40 μM inhibitor for 16 h, bar = 100 μm, **(C)** the detection diagram of IMP content by HPLC; **(D)** the effects on the expression of AMPK-mTOR signaling pathway related genes. Error bars represent the means ± SD; *n* = 3. *p* < 0.05.

In order to clarify the effect of *AMPD1* on IMP formation, we first verified it by *in vitro* experiments. We first established triploid crucian carp muscle cells *in vitro* (Figure 7A), and we added different concentrations of AMPD1 inhibitor (0, 20, 40, and 60 μM) into the sixth-generation muscle cells for 16 h to detect the expression of IMP. The sixth generation of muscle cells grew well after being treated with 40 μM AMPD1 inhibitor for 16 h (Figure 7B). And the results showed that the IMP content in muscle cells after 40 μM AMPD1 inhibitor were significantly reduced compared with the control group (Figure 7C). In order to preliminatively clarify the regulatory mechanism of *AMPD1* regulating IMP formation, The effects of AMPD1 inhibitor on the expression of AMPK-mTOR signaling pathway related genes in triploid crucian carp muscle cells cultured with 40 μM AMPD1 inhibitor for 16 h were further examined by Q-PCR at the cellular level. The results showed that 40 μM AMPD1 inhibitor promoted the expression of *AMPK* in muscle cells cultured for 16 h and inhibited the expression of *AMPD1*, *mTOR*, and *p70S6k1* genes (Figure 7D).

## Discussion

Meat quality is an important economic trait that includes texture, nutrition value, flavor, etc, and flavor is closely related to the muscle IMP content (Yan et al., 2018; Zhu et al., 2021). *AMPDl* gene is one of the key enzymes in purine nucleotide cycle. It is responsible for the catalytic cleavage of AMP to IMP, and plays a very important role in energy metabolism of eukaryotes (Yu et al., 2021). Studies have shown that *AMPD1* gene is mainly expressed in animal muscle, and it is the key enzyme of AMP generating IMP in animal muscle, and plays a decisive role in muscle flavor (Takayanagi et al., 2020). In this study, *AMPD1* was detected in seven different tissues of triploid crucian carp, the results showed that *AMPD1* was mainly expressed in muscle. At present, the research on *AMPD1* gene in mammals and poultry mainly focuses on cloning *AMPD1* gene sequence and analyzing the relationship between *AMPD1* gene polymorphism and IMP content (Norman et al., 1998; Norman et al., 2001; Hu et al., 2015). And most current studies only study the correlation and polymorphism of *AMPD1* gene and IMP content, in particular, the molecular regulatory mechanism of *AMPD1* regulating IMP formation has not been studied yet, and there are few studies on *AMPD1* gene in aquatic animals. IMP is also known as hypoxanthine nucleotide, is one of the main components of muscle umami and fragrance. IMP content has been regarded as an important index to measure meat flavor in the world (D. Li et al., 2017; Takayanagi et al., 2020). Studies have shown that IMP is a major contributor to meat flavor in livestock, poultry and fish muscle, and its ability to increase umami taste is tens of times stronger than that of glutamate (Bonagurio et al., 2022). Therefore, the flavor of livestock, poultry and fish mainly depends on the content of IMP in meat. The mechanism of IMP formation after slaughter is that ATP in muscle is decomposed into ADP under the action of ATPase, and then ADP is decomposed into AMP under the action of creatine kinase, and then AMP is formed into IMP under the action of *AMPD1*. Mammalian studies have shown that down-regulation of AMP levels can regulate biological energy metabolism by altering AMP protein kinase (AMPK) activity. AMPK widely exists in various eukaryotic cells and is an important cell energy receptor. AMPK can sense AMP: ATP and ADP: ATP ratio changes and is activated, by inhibiting the anabolic process that consumes ATP, promote the catabolic process that produces ATP, thus restoring energy balance. After activation of AMPK, ATP production is increased and ATP consumption is decreased by increasing glycolysis, glucose uptake and inhibiting protein synthesis, which directly affects the formation of IMP (Cheng et al., 2014; Vignozzi et al., 2014; Yi et al., 2015). Studies also have showed that feed composition can affect the evaluation result of muscle IMP (Dyachok et al., 2016; Hossain et al., 2019). Glutamate was an important flavor amino acid, and the results had showed that dietary glutamate significantly increased the IMP content in muscle of prawn. In addition, studies on yellow-feathered broilers showed that dietary glutamate could improve the content of flavor amino acids in muscle and the expression of genes related to the formation of flavor substances, so as to improve meat flavor. In fish studies, it was found that dietary glutamate could improve the muscle fiber structure and muscle texture of grass carp, and increase the content of IMP in muscle (Pan et al., 2016; Jiang et al., 2017; Tan et al., 2017). However, these studies only described the phenomenon of amino acid regulation of IMP formation, and almost did not involve the metabolic process and regulatory mechanism. Therefore, it is necessary to study the molecular basis of amino acid metabolism and regulation of IMP formation in fish. In conclusion, in this study, we cloned the full ORF sequence of *AMPD1* gene by homologous cloning method for the first time in triploid crucian carp and analyzed the cloned *AMPD1* sequence using bioinformatics techniques, and we analyzed the expression of its organizational characteristics. And we examined the effects of glutamate which was the promotor of IMP formation on the expression of *AMPD1* and the formation of IMP *in vivo* and *in vitro* experiments, the results showed that 3% glutamate and 2 mg/ml glutamate could significantly promote *AMPD1* expression and IMP formation in triploid crucian carp muscle tissue and muscle cells (*p* < 0.05). Further we studied *AMPD1* gene function through *in vivo* and *in vitro* experiments, and we proved that *AMPD1* played a key role in the process formation of IMP. And the results showed that its regulatory mechanism was inhibition of *AMPD1* inhibited AMP to form IMP, increased AMP and promoted *AMPK* expression, and then inhibited *mTOR* and its downstream *p70S6K1* expression, and finally inhibition of protein synthesis pathway reduces ATP consumption and further reduces IMP formation. This study confirmed that *AMPD1* mediated glutamate through AMPK-mTOR signaling pathway to regulate the formation of fish muscle IMP. Therefore, this study will provide a theoretical basis for establishing a reasonable feeding strategy for cultured fish and improving the umami quality of cultured fish, and it will put forward a new idea for in-depth study on accurate nutrition regulation of fish quality traits.

## Data Availability

The datasets presented in this study can be found in online repositories. The names of the repository/repositories and accession number(s) can be found below: https://www.ncbi.nlm.nih.gov/, MW435570.1.
